# Beyond the review: additional non-oncologic drugs with clinically significant cardiovascular effects

**DOI:** 10.1093/ehjcvp/pvag029

**Published:** 2026-04-24

**Authors:** Lorenz Van der Linden, Thomas Vanassche

**Affiliations:** Hospital Pharmacy Department, University Hospitals Leuven, Herestraat 49, Leuven 3000, Belgium; Department of Pharmaceutical and Pharmacological Sciences, KU Leuven, Herestraat 49, 3000 Leuven, Belgium; Department of Cardiovascular Sciences, KU Leuven, 3000 Leuven, Belgium; Department of Cardiology, University Hospitals Leuven, 3000 Leuven, Belgium

**This correspondence refers to ‘Cardiovascular adverse effects of common non-oncologic medications: from mechanisms to clinical management’, by M.C. Bahit et al., https://doi.org/10.1093/ehjcvp/pvag007.**

The review by Bahit *et al*. provides a comprehensive overview of the cardiovascular risks associated with non-oncologic medications.^1^ By bridging molecular mechanisms with clinical management, the authors provide a valuable resource for managing complex patients. To further enhance the scope, several additional drug classes with distinct and significant cardiovascular profiles warrant consideration, particularly in specialty care settings.^2,3^

As a class, azole antifungals share a propensity for QTc prolongation, except for isavuconazole, which rather shortens the QT interval and is contraindicated in familial short QT syndrome.^4^ In the context of medication harm, itraconazole represents a unique case of direct cardiovascular toxicity.^5^ Unlike most agents that cause harm via secondary pathways, it exerts direct negative inotropic effects and is formally contraindicated in patients with ventricular dysfunction. Its inhibition of CYP3A4 further amplifies the risk of interactions with co-administered cardiovascular drugs. The antifungals voriconazole and posaconazole are potent CYP inhibitors as well, though with distinct profiles: voriconazole broadly inhibits CYP3A4, CYP2C9, and CYP2C19, whereas posaconazole primarily inhibits CYP3A4 with substantially less effect on CYP2C9 and CYP2C19. These pharmacokinetic properties can lead to dangerous elevations in plasma concentrations of statins, antiarrhythmics, and calcium channel blockers, often precipitating QTc prolongation or profound bradycardia.^6^ Even fluconazole, the most frequently prescribed azole, may appear to carry the mildest individual risk profile among the azoles, yet its ubiquity across virtually all specialties translates into a disproportionately high population-level burden that no prescriber should overlook.

The relevance of CYP-mediated interactions is shaped by three factors: (i) potency of the culprit agent at clinically relevant exposures, (ii) degree of metabolic redundancy of the substrate, and (iii) the therapeutic margin of the affected drug.^7^ Drugs with several parallel clearance pathways may therefore tolerate inhibition of a single CYP isoform with only modest changes in exposure.^7^ The potent triazoles highlighted here, however, represent situations in which clinically meaningful interactions are more likely. Many susceptible substrate drugs have limited alternative metabolic pathways, and substantial increases in exposure of statins, calcium channel blockers, and several antiarrhythmics are well documented.^8^

The cardiotoxicity of domperidone, a peripheral dopamine antagonist widely used for gastroparesis and nausea, also merits attention. Domperidone is a hERG channel blocker associated with dose-dependent QTc prolongation and an increased risk of sudden cardiac death, particularly at higher doses and in older adults.^9^ Critically, its primary metabolism via CYP3A4 means that co-administration with azole antifungals can substantially increase domperidone plasma concentrations, compounding arrhythmic risk even further.

Methadone likewise requires careful monitoring. As a potent hERG channel blocker, it causes dose-dependent QTc prolongation and increases the risk of torsades de pointes.^10^ Its long and highly variable half-life complicates risk assessment, particularly when co-administered with other QTc-prolonging agents mentioned in the review, such as macrolides or fluoroquinolones.^1^ This compounded arrhythmic risk is important to cardiologists consulting on patients in addiction or chronic pain services, where such interactions may be underappreciated.

The cardiovascular risk profile of combined oral contraceptives and progestins also deserves attention. Beyond the well-recognized risk of venous thromboembolism, these agents influence arterial thrombotic risk, including stroke and myocardial infarction. Recent evidence indicates that while absolute risks remain low, contemporary combined formulations are associated with a roughly two-fold increase in the risk of ischaemic stroke and myocardial infarction compared to non-use.^11^ The underlying mechanisms, including oestrogen-driven upregulation of coagulation factors and progestin-mediated effects on vascular tone and lipid profiles, align well with the mechanistic focus of the authors’ review.

Two further agents merit consideration. Romosozumab, a sclerostin inhibitor for severe osteoporosis, is contraindicated in patients with a history of myocardial infarction or stroke. Its growing use in older adults with substantial cardiovascular comorbidity means that awareness of the risk profile cannot be overstated.^12^ Janus kinase inhibitors, including tofacitinib and upadacitinib, carry boxed warnings for major adverse cardiovascular events, venous thromboembolism, and heart failure, particularly in patients aged 65 years or older or in those with established cardiovascular disease or risk factors.^13^ These therapies, largely initiated outside cardiology, demand multidisciplinary familiarity with their cardiovascular safety profile.

While every review must be selective, incorporating these agents into future updates or companion practical documents could further strengthen its utility for the multidisciplinary teams.

## Data Availability

No data were generated or analysed for this article.
